# Bio-chemical and molecular analysis in cardiomyopathy patients

**DOI:** 10.1186/1755-8166-7-S1-P73

**Published:** 2014-01-21

**Authors:** Rutvik J Raval, Tapan A Patel, PL Sachapara, Mandava V Rao

**Affiliations:** 1 Gujarat Genetic Diagnostic Center (GenDiCe), Department of Zoology, University School of Sciences, Gujarat University, Ahmedabad, Gujarat, India; 2 Samarpan Hospital, Madhavjyot, Kalubha road, Bhavanagar, Gujarat, India

## Introduction

Cardiomyopathy refers to diseases of the heart muscle. Symptoms of cardiomyopathy includes: Shortness of breath, Chest pain, Dizziness, Lightheadedness or fainting, Palpitations. Cardiomyopathies are divided in two types that are Primary (intrinsic) and Secondary (extrinsic) cardiomyopathies. Primary cardiomyopathies are divided in three types i.e. genetic, mixed and acquired.

Present study was planned to evaluate the biochemical alterations in patients with cardiomyopathy or myocardial Infarction using bio-chemical and molecular indices.

## Methods

Blood samples were collected from patients (n=12) with cardiomyopathy/myocardial infarction and same age and sex matched healthy controls (n=12). Serum myoglobin and serum creatine kinase-MB (CK-MB) levels were evaluated. Genomic DNA was isolated from peripheral blood and its quality and quantity were checked. PCR amplification of the various exons of genes MYH7 (exon 8 and 9) and *TNNT2* (exon 8) was carried out. PCR products were checked by 2% agarose gel electrophoresis. Further, mutations were screened by Polymerase Chain Reaction-Single Strand Conformation Polymorphism (PCR-SSCP) technique.

## Results

In cardiomyopathy patients the ratio of Male: Female was 1:1. Out of twelve patients four patients showed high levels of CK-MB and two patients with high Myoglobin levels and only one patient reported high levels of both myoglobin & CK-MB. None of the patient showed mutation in exon8 and 9 of MYH7 gene & exon 8 of *TNNT2* gene.

## Conclusion

In Gujarat population, cardiomyopathy patients exhibited alterations in biochemical parameters, but did not reveal any mutation in exon 8 and 9 of MYH7 gene &exon 8 of *TNNT2*.

